# ‘*I*’*m getting the balls to say no*’: Trajectories in long-term recovery from problem substance use

**DOI:** 10.1177/1359105320941248

**Published:** 2020-07-21

**Authors:** Lucy Webb, Amanda Clayson, Eva Duda-Mikulin, Nigel Cox

**Affiliations:** 1 Manchester Metropolitan University, UK; 2 VoiceBox Inc., UK; 3 University of Bradford, UK

**Keywords:** community health psychology, health psychology, public health psychology, relapse prevention, social capital, social interaction, social network, substance abuse

## Abstract

This study uses individualisation theory to explore identity transition in substance misuse recovery. Identity narratives gained over 4 years from co-produced video/audio interview and video diary accounts were co-productively collected and analysed using framework analysis. Results indicate a trend towards individualistic and agentic identity as recovery trajectories progress over time. Within-case analysis demonstrates agentic growth for most participants, from early-stage gratitude and reliance on support groups to self-determination and independent decision-making. This early work exploring longer-term recovery adds to the current recovery and social identity discussion and provides evidence of identity growth in longer-term stages of recovery.

## Introduction

An important aspect of substance use recovery research explores how development of positive social identity supports people through the recovery process and protects against relapse (Best et al., 2014; 2018; Longabaugh et al., 2010). However, little evidence focuses on identity in longer-term recovery or which identity elements assist in maintaining lifestyle change.

This article reports the first application of a model of developmental individualisation (Schwartz et al., 2005) to explore identity development in sustained recovery from problem substance use (alcohol and illicit drugs) to map identity change along the recovery trajectory. It therefore opens an important extension to understanding how people maintain behaviour change and what practitioners need to consider in supporting people towards longer-term recovery.

Using framework analysis (Ritchie and Spencer, 2002), we use indicators of identity from recovery narratives to construct themes of recovery identity, and map these identity themes onto stages of the individualisation model. Finally, the analysis maps stages of individualisation onto stages of recovery, by early, mid-stage or longer-term recovery.

The data and the analysis are embedded in a co-productive methodology as part of the ‘Voices from the Brink’ project (described below) to explore lived experience of recovery over time (Clayson et al., 2018). The aim of the analysis is to establish whether there are maturation processes that correlate with recovery over time, moving from a social identity reliant on external factors of group validation and support, towards an agentic and individual identity that is supported by factors of internal resilience.

### Social identity and recovery

A key factor shown to aid people transitioning from substance misuse to recovery is belonging to mutual aid recovery groups (Beckwith et al., 2019; Humphreys and Lembke, 2014). Buckingham et al. (2013) argue that social identity theory may be useful in explaining why some individuals relapse while others remain in recovery. Preference for identity associated with recovery, rather than addiction, may increase specific self-efficacy beliefs and be associated with new behaviours (Tarrant and Butler, 2011). During group therapy and mutual aid meetings, a new identity focused on ‘recovery’ will be developed – a ‘recovering addict’ identity compared to the existing ‘addict’ identity (Orford, 2001). Beckwith et al. (2019) demonstrate that the strength of the ‘recovery identity’ is associated with the proportion of non-substance-using social networks. Even when not immediately able to leave their ‘addict’ identity, people in recovery may draw on their ‘recovery identity’ to avoid relapse. The process of gradually moving away from the ‘addict’ identity, towards the ‘recovering addict’ identity, has been termed ‘identity preference change’ (Buckingham et al., 2013).

Johansen et al. (2013) assert that community recovery networks can be more effective than professional treatment. They argue that social support programmes should be based on recovery networks, which can result in increased self-esteem and, essentially, identity change. They identify effective recovery-related processes include positive identity-building, perceived support and control, and self-verification of positive identity. Their findings demonstrate that social support should aid the person to develop a positive self-image to enable recovery.

Mawson et al. (2015) stress the need to consider the type and composition of social networks conducive to recovery. They differentiate recovery capital between social capital, comprising supportive social groups and family relationships, and personal capital which encompasses material resources (i.e. finances, housing) and intra-personal capital (i.e. self-esteem, motivation). For Mawson et al. by changing the social environment and associating with non-using groups, the recovering individual can develop an identity associated with recovery rather than substance use. Indeed, clinical research indicates that social networks that support abstinence are associated with reduced risk for relapse (Litt et al., 2009; Zywiak et al., 2009).

Best at al. (2014) also underline the importance that recovery capital has in the development of a social identity other than ‘substance user’. For Best et al. (2014, 2018) and Jetten et al. (2012), strong social networks and a social identity confer a form of capital that enables further development of group and social identity.

### Identity development theory, identity capital and individualisation

The notion of identity change in recovery resonates with identity development theories whereby identity evolves and maturates throughout the lifespan. Erikson’s eight-stage model (1968) and Marcia’s (1966) four-status model are based on epigenetic identity development: a transitional concept of development in which identity is shaped on the basis of past identity and interaction with the environment. Epigenetic identity theory explains identity capital as representing the person’s internal resources, interacting with external environmental resources (Schwartz et al., 2005). In recovery these may present internally as self-esteem and motivation, and externally as treatment access, mutual aid groups, employment or non-using social support (Côté, 2002).

Côté’s (2002) theory of identity capital uses epigenetic developmental theories to propose identity development as a series of transitional processes, moving from a passive default position of an externally conferred ‘given’ identity (i.e. from family and culture) to an internalised ‘agentic’ position of individuality. According to Côté, agentic identity is acquired through interaction with new environments, through exploration, risk-and opportunity-taking, resulting in a new sense of self and agency separate from the previous ‘given’, or, for Côté, passive, identity. This transitional process is facilitated by social support, community membership, validation from others and having opportunities for exploration. The result of this process is stable maturity, resilience (ego strength, confidence, purpose) and adaptability, enabling self-determination and personal choice. Importantly, agentic identity is more individualistic than a social identity that is supported by group identification and validation from external forces such as family, friends and culture.

**Figure 1.**
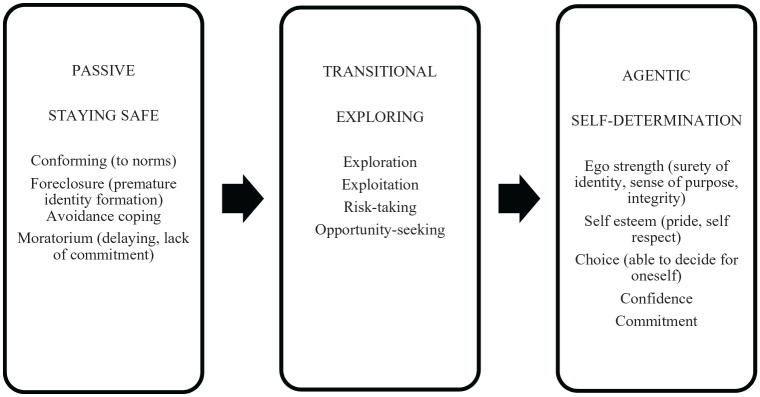
Framework adapted from Côté’s individualisation hypothesis and Schwartz et al. Agency-identity model.

In epigenetic development theory, the process of identity maturation is mediated by over- or under-exposure to opportunities for self-determination and exploration, where under-exposure can cause frustration and timidity, and over-exposure leads to fear of failure and negative or stressful experiences (Côté, 2002; Luyckx et al., 2011). Both may result in a passive identity; lack of confidence, reliance on labels and others’ validation, or avoidance behaviour (Schwartz et al., 2005). For Schwartz et al. (2005), passive identity is epitomised by conformity, foreclosure (premature identity formation), avoidance and lack of commitment, while agentic growth is epitomised by self-exploration, risk-taking, and exploitation of opportunities. Agentic growth results in positive self-esteem, ability to make independent choices, a sense of purpose and commitment (Figure 1).

The evidence for the importance of social identity for those in recovery has tended to focus on people in early stages of recovery in which they are transitioning from an ‘addict’ identity to ‘in recovery’. This study explores longer-term recovery to focus on what identity elements sustain recovery and support maintenance of healthy lifestyle change. To do this, we examined identity change over time, from short-term to longer-term recovery, to explore whether identity change progresses along the recovery trajectory from social identity towards individual identity.

## Aims of the study

The aim of this study was to use individualisation theory to explore transitions of identity over time among people in substance use recovery, from immediate treatment exit to up to 4 years in recovery. Specifically to:

1. Identify and explore identity changes over longer-term recovery;
2. Explore individualisation theory in explaining identity changes and resilience in longer-term recovery and so identify support needs in sustaining recovery.

## Method

This study applied framework analysis to data from video and audio interview and diary-style narratives, recorded by people on substance use recovery trajectories. This material represents cross-sectional sampling of narratives relating to recovery experiences, from treatment completion to 12 months post-treatment (early-stage recovery), 18 months to 2 years post-treatment (mid-stage recovery), and 3–4 years post-treatment (later-stage recovery). Co-production was embedded across the research process, from conception through to qualitative analysis, compatible with ‘INVOLVE’ guidelines for inclusive research (Hickey et al., 2018). These guidelines ensure that participants are fully included in the research process equally and their perspectives and knowledge is respected and valued. Co-production offers an overarching methodology that therefore adds to the credibility, confirmability and dependability of qualitative approaches (Durose et al., 2017) and reduces elitist narrative (Crompton, 2019) that may carry an academic bias in qualitative interpretation.

Ethical approval was obtained from the university ethics committee for the ‘Voices from the Brink’ project of which this study is a part (see ‘*Data collection*’). To protect participants, pseudonyms have been applied, and non-relevant details altered to avoid the deductive identification of participants (Saunders et al., 2015).

### Participants

Narratives from six participants were purposively sampled from a wider archive of collected video and audio material (30+ individuals), captured at different stages of their recovery trajectories. Participants were selected on the basis of each having captured narratives for all stages of recovery (from early-stage to 3+ years post-treatment), and representing a range of substance-use experiences and demographics. All participants were from the North West of England, having received either community and/or inpatient treatment for problem substance use, following a 12-step treatment or a combined cognitive-behavioural and rational emotive approach called ‘SMART’ (self-management and recovery training) (Table 1).

**Table 1.** Participant characteristics.

Pseudonym	Gender	Approximately age of recovery commencement	Treatment type received	Substance used
Terry	M	50	SMART	Alcohol
Len	M	45	12 step	Alcohol
Liz	F	39	12 step	Drugs and alcohol
Gary	M	27	12 step	Drugs
Barbara	F	40	SMART	Drugs
Ron	M	38	12 step	Drugs and alcohol

### Data collection

Recorded material is part of an ongoing longitudinal community participatory research project, ‘Voices from the Brink’, exploring and capturing people’s experiences of recovery from problematic substance use. Data were collected largely by video interview via the Voicebox interview booth (see Cox et al., 2016), or by participants’ own video diaries shared with the project. Interviews took place during a range of recovery-oriented events between 2014 and 2017 (i.e. Recovery Walk UK, NHS Expo) and pre-arranged personal interviews. Video interview data were collected to elicit experiences of being in recovery, exploring what recovery means to interviewees and the impact of being in recovery. By later-stage recovery, participants had also viewed an ‘early-stage’ interview of themselves and recorded their reflections on their personal change. Additionally, video diaries were offered to the project by several participants on an ad hoc basis throughout the stages, and one audio interview was included. Overall, 29 separate narratives were included in the analysis.

### Analysis

Framework analysis was adopted as it facilitates the use of deductive a priori theory (Ritchie and Spencer, 2002), namely, the individualisation theory in this study. Framework analysis is particularly suited to exploring cross-sectional qualitative data (Ritchie et al., 2013), and provides a sequential process to data management and interpretation (Hackett and Strickland, 2018). It offers a structured thematic framework to cross-reference themes and categories and facilitates within- and between-case comparisons (Gale et al., 2013). The individualisation model stages were given super-ordinate themes of ‘staying safe’, ‘exploring’ and ‘self-determination’ (Figure 1) to simplify the initial coding process.

#### Reflexivity and positionality

The cultural competence (Case, 2017) of the community partner to this research served to strengthen the credibility of the qualitative analysis; nonetheless, critical attention was paid throughout the research process to potential issues relating to reflexivity. For example, whilst her ‘insider-outsider’ positionality arguably improved the credibility of analysis (by way of her cultural competency), her position may have also conferred upon analysis the ‘insider complacency’ and loss of dependability noted by other researchers using co-production approaches (Gormally and Coburn, 2014). The authors, including the community partner, have previously published a detailed account of their general approach to supporting reflexivity (Clayson et al., 2018).

#### Framework analysis

Typically, framework analysis comprises seven stages (Gale et al., 2013); for clarity, the analysis combined these into four stages (Gale et al.’s stages in parentheses):

1. Identification of identity statements (Transcription, Familiarisation)

Working from video and audio material directly, familiarisation involved reviewing all included material to become immersed in the data. This stage resulted in an understanding of interviewees’ expressions of identity, and contexts of the narratives.

2. Thematic analysis of statements and framework development (Coding, Identifying the thematic framework, Indexing: applying the framework)

Reviewed material was coded into themes. Although this study applied a deductive approach, themes were not pre-set but merely guided by the three super-ordinate themes of the individualisation model. Therefore, coding remained open and subject to inductive, emergent identification of these governing themes.

Themes (codes) were then grouped into categories to create the thematic framework. Ritchie and Spencer (2002) emphasise that this stage requires intuitive and reflexive thinking and it was noted that co-productive working improved understanding of the recovery contexts and language used by participants (see ‘interpreting the data’ below). This stage resulted in a thematic framework relating to identity development and patterns of identity expression from the participants (Table 2). These themes were then compared with the original statements to ensure rigour.

**Table 2.** Thematic framework matrix.

Super-ordinate theme (stage of individualisation)	Themes from identity statements	Categories (collapsed themes)
Staying safe (Passive)	Gratitude	Gratitude
	Feeling lucky	
	Validation	Needing
	Belonging	
	Scared	
Exploring (Transitional)	Feeling exposed	Taking risks
	Learning about self	
	Helping others	
	Frustration	Seeking opportunities
	Connecting with people	
Self-determining (Agentic)	Looking after self	Integrity and purpose
	Making own decisions	
	Confidence	Self-worth and belief
	Pride and respect	
	Acceptance	‘The real you’
	Authentic

3. Statements mapped onto individualisation grid by stage of recovery (Charting)

Cross-referencing of themes and categories (charting) performs a validation check and resulted in the framework matrix of all participants’ transcript material (Table not shown). For our study, the framework was then added to the recovery stages and enabled analysis of trends of identity change over time as categorised by ‘early’, ‘mid’ and ‘later’ recovery stage (Supplemental Table 1).

4. Interpreting the data

Interpretation runs parallel with all stages. Gale et al. (2013) recommend noting down impressions and ideas during early analysis, and to explore such material with the research team or lay members. In line with the co-productive approach, this was applied and found helpful in identifying additional overarching themes that may not otherwise have been identified, here termed ‘Fellowship speech’. Fellowship organisations (i.e. Alcoholics Anonymous) use specific phrasing to describe abstinence and/or recovery experiences, encourage giving gratitude to others and acknowledging a ‘higher power’. It was observed that participants often made selections from this linguistic repertoire when describing their recovery; their use of such ‘Fellowship speech’ informed interpretation, though the theme was not included in the framework.

## Results

Initial thematic analysis resulted in 16 themes guided by individualisation theory, and framework development produced six categories from the themes to form the framework matrix (Table 2).

Charting cases to the framework matrix resulted in few empty cells, with everyone making passive, transitional and agentic statements. Empty cells were evident in the final chart for most participants when mapping the divisions of ‘early’, ‘mid’ and ‘later’ stage of recovery by case, with trends towards agentic growth in later stages. For this study, empty cells would be expected where maturation developed in line with length of recovery (i.e. creating a positive correlation pattern) (Supplemental Table 2).

### The individualisation model in recovery trajectories

#### Passive – Staying safe

Key themes coded as ‘gratitude’ suggested humility at ‘being saved’ and feeling indebted to external factors; organisations, recovery groups or fate. Expression of these statements often had a feeling of desperation; like clinging to a life-raft. This was present for all informants across stages of recovery but most typically in the early-stage:

*Glad to be here. I’m lucky I’ve got a roof over my head* (Ron, early-stage).

*If it hadn’t been for (treatment centre) I wouldn’t be a triathlete* (Len, early-stage).

Neediness emerged from a need to be with others, especially when feeling vulnerable, to obtain motivation and validation of belonging. Participants all demonstrated gratitude or humility and made various statements of dependence and reliance on support groups or communities in both the early- and mid-stages of recovery:

*Having somewhere to be at a specific time (to mutual aid group) benefits me a lot* (Ron, early-stage).

Feeling threatened was also evident in early- and mid-stage accounts:

*We need people to help us along the way. . . not be pushed* (Terry, mid-stage)

*. . . for them (work assessors) to tell me if I’m well enough to work . . . . (made me) really anxious (doesn’t feel up to working yet but will lose benefits).* (Barbara, mid-stage).

Validation from others, and identifying/belonging with others in recovery was important in the early stage but there were no statements from this theme in mid or later stages:

*They (non-addicts) are not like us* (Liz, early-stage)

*I just hoped to gain some more confidence (out of the group)* (Len, early-stage)

*Feel a little crap about things. Going to (recovery group). Can only get better today* (Barbara, early-stage).

#### Transitional – Exploring

There were instances of explorative behaviour and risk-taking throughout the stages, but particularly at the early- and mid-stages. Many spoke of exploring or discovering more about themselves, discovering ‘old’ selves, and discovering new skills and strengths:

*I had the opportunity to (do) something I used to enjoy. Made me feel less anxious. I was a bit nervous just (doing) something I have done before* (Gary, early-stage)

*I come to groups to volunteer. It’s liberating* (Liz, early-stage)

*The smile is real, loving it. Re-discovering life, finding myself* (Barbara, mid-stage)

*I just learned about my own wellbeing and confidence. I’ve learned I’ve got choices* (Len, mid-stage).

Frustration at not being able to explore was also evident, suggesting a development need to ‘move on’ and enter a transitional stage. This was evidenced in several expressions of frustration of being ‘held back’, being bored or obstructed by barriers. Terry’s impassioned expression, ‘*we*’*re not all useless*’ (mid-stage), felt like frustration at not being allowed to contribute to work (he appeared to place a lot of self-worth in employment), and Barbara’s frustration at being stuck in her flat because of illness and saying ‘*I*’*m bored, bored, bored*’ (later-stage) came over powerfully in the video source.

There were instances where people withdrew from over-exposure to transitional opportunities, feeling threatened by uncontrollable change. Barbara’s expressed fear at mid-stage (above) that she might be told she was fit for work was expressed forcefully in her video diary and it was clear that she felt she was being pushed too fast towards self-reliance.

However, being scared or feeling anxious about change was also often met with determination and risk-taking:

*I was a little anxious if I’d understand [the recovery training course], worried if it was the right thing for me* (Liz, early-stage)

*I now deliver a self-help group. That’s a big step for me* (Len, mid-stage).

#### Agentic – Self-determination

Statements of agency and personal integrity predominated in the mid and later recovery stages. One participant (Liz) had only one entry in a self-determination cell by later-stage but all other participants expressed a need or willingness to make their own decisions:

*I made a brave decision to become what I wanted* (Gary, mid-stage)

*I think I have become comfortably uncomfortable* (Ron mid-stage)

*. . . (I) asked myself, what does ‘looking after (me)’ look like? So I said to the lady (at the volunteering centre) I’m not coming in today. I’m going to my friend’s, do a bit of gardening which I enjoy*. (Barbara, later-stage).

*Recovery is when you are on your own, making your own decisions. I’m getting the balls to say ‘no’*. (Terry, later-stage).

Early stages of self-determination feel less authentic and more like ‘flight to health’ experiences:

*It’s good to have feelings* (Liz, early-stage)

*Just be the real you for a change* (Barbara, early-stage).

However, expressions of agency and personal integrity were notable in the later-stage for several participants who expressed attitudes of distancing from groups and of independence:

*I can’t think of one negative thing. I don’t need to. [ ] I’ve met a nice girl. Good luck to everyone. I hope everything pans out for everyone, but I can only look out for me and (my family)* (Ron, later-stage)

*Recovery, they (people generally) should call it something else – it’s not recovery it’s living again* (Terry, later-stage)

*For me, it’s my own personal journey. It’s about discovering who I am* [ ] *(gave up volunteering, felt exploited, got a job and focuses on triathlon now – report to interviewer after recording)* (Len, late-stage).

## Discussion

The overall aim of this study was to use individualisation theory (Côté, 2002; Schwartz et al., 2005) to explore identity change among people transitioning from early to later stages of recovery. Within-case analysis demonstrates agentic growth for all but one participant, with a trend moving from early-stage gratitude and dependence, through exploring and risk-taking, to later distancing from fellow recovery members, and on to an apparent self-possession and independence.

### Environment and context of recovery

All participants had experienced formal substance use treatment programmes and were engaged in post-treatment recovery activities such as volunteering as ‘recovery champions’, attending mutual aid groups or taking a social development course. Therefore, recovery activities are contextual factors for all participants. The influence of these activities is likely to be associated with the engendered sense of belonging and identification with ‘being in recovery’ as suggested by Buckingham et al. (2013). These contexts have provided identity growth opportunities, such as the development course, recovery events, meeting people and helping others. Additionally, these participants were engaged in the ‘Voices from the Brink’ project which encouraged self-reflection on recovery identity.

The interpretation stage indicated Fellowship influences in many statements. These were particularly strong and unchanging for Liz who makes mostly social identity statements and few statements of agency or self-determination throughout her recovery trajectory. Fellowship concepts of gratitude and helplessness convey a de-powering of the self, and influence ideas of powerlessness, or ‘*higher power*’ (Gary, mid-stage). The Fellowship context of recovery facilitates change in social networks and improves social factors that support abstinence (Kelly et al., 2011). In this analysis, its concepts appear utilised as elements of a newly ‘given’ identity, and are features that appear particularly strong in the earlier stages of recovery for all participants.

### Identity transition and recovery

While much research rightly focuses on what enables people with addictions to engage in behaviour change, evidence of sustained personal change tends to be focused on the continuing role of recovery group support for relapse prevention (Kelly et al., 2011), or what recovery as a community phenomenon may have on longer-term change (Best et al., 2018; Humphreys and Lembke, 2014). The results from mapping maturation on to stages of recovery indicate a slope that suggests a distancing from recovery identity in later stages of recovery, and this appears to be epitomised by identity growth from a social identity to an individual and agentic identity. These findings suggest that there may be trends towards individualisation in later stages for some people. Evidence is found here of a movement away from social identity to a growing exploration in mid-stage and on to an ability and willingness for self-determination.

Recovery identity change appears compatible with the individualisation model developed from epigenetic identity theory. It enables an understanding of the interaction between internal identity needs and external resources; how social networks support social identity and how opportunities such as post-treatment training or re-engagement with non-using networks may enable internalisation of identity strengths. This mapping may indicate that mutual aid recovery engenders the maturation process among adults with substance use problems. It is important however to recognise that social identity appears to be important for the transition from ‘addict’ identity to early-stage recovery, and these findings support that social identity, characterised here by a need to belong and be validated externally, is an important factor in early-stage recovery. Also, as evidenced by Liz in this study, social identity and continuing mutual aid support is a vital element in sustaining recovery for some people. This early work in exploring longer-term recovery however may provide an explanation of identity growth further along the recovery trajectory. While personal growth is aligned with resilience, it remains to be determined if individualisation is as protective of abstinence maintenance as having a positive social identity.

## Strengths and limitations

This is a small study of multiple narratives from six participants and can only indicate if some people may experience identity change over time. If recovery and maturation are linked, it cannot be determined from this methodology if addiction recovery or natural processes can account for the development of agency, nor indeed if the development of social identity in recovery is a necessary step towards agentic identity.

While the small sample and lack of a control group are limitations, the naturalistic data elicited from open interviewing and self-generated diary posts reduces researcher bias by an absence of pre-determined questioning. Also, the use of video-as-transcript aided interpretation through access to the non-verbal communication, while the co-productive nature of the overarching methodology enabled a ‘discursive legitimacy’ (Purdy, 2012) to the interpretation of the material and its meaning.

## Implications

For practitioners supporting people in recovery, a maturational shift for people sustaining recovery in the longer term has implications when assisting maintenance of behaviour change. The transtheoretical model (Prochaska et al., 1992) highlights maintenance for people in control of their substance use. Current evidence supports the role of social identity in supporting early-stage recovery but in later stages there may be a need to make use of opportunities to develop a sense of independence, and improve self-esteem through further achievement. It is important that factors supportive of later-stage recovery maintenance are explored to facilitate timely personal development of internal resilience.

For researchers, both academic and community-based, this study also demonstrates the vitality and fidelity of co-production research methodologies for exploring substance use recovery narratives. Choice of material, identification of themes and interpretation of meaning all relied on understanding the nature of ‘being in recovery’ and the context of the given narratives. Ethnographic and reflexive methods, as used here, support co-production principles in that they recognise that participants and partners possess material and cultural knowledge by way of their lived experiences. This knowledge is instrumental in the identification and amelioration of the methodological challenges of research with a ‘seldom heard’ population (Clayson et al., 2018). Moreover, contrary to conventional research methodologies, co-productive research, supported by framework analysis, has supported the authenticity of the findings.

## Conclusion

Identity change is an important research concept where community and psychosocial elements of recovery are becoming recognised as key factors in sustaining abstinence. A larger cohort of participants and a longer recovery trajectory (5 years+) would better test maturation processes and ascertain whether growth away from social identity increases or decreases risk of relapse in later life.

## Supplemental Material

Supplementary_Table_1 – Supplemental material for ‘I’m getting the balls to say no’: Trajectories in long-term recovery from problem substance use Click here for additional data file. Supplemental material, Supplementary_Table_1 for ‘I’m getting the balls to say no’: Trajectories in long-term recovery from problem substance use by Lucy Webb, Amanda Clayson, Eva Duda-Mikulin and Nigel Cox in Journal of Health Psychology

Supplementary_Table_2 – Supplemental material for ‘I’m getting the balls to say no’: Trajectories in long-term recovery from problem substance use Click here for additional data file. Supplemental material, Supplementary_Table_2 for ‘I’m getting the balls to say no’: Trajectories in long-term recovery from problem substance use by Lucy Webb, Amanda Clayson, Eva Duda-Mikulin and Nigel Cox in Journal of Health Psychology
