# Supplementary material for: ‘I’m getting the balls to say no’: Trajectories in long-term recovery from problem substance use
Source: J Health Psychol. 2020 Jul 21;27(1):69–80. doi: 10.1177/1359105320941248 (PMC8739601; doi:10.1177/1359105320941248)
Supplement: Supplementary_Table_1 – Supplemental material for ‘I’m getting the balls to say no’: Trajectories in long-term recovery from problem substance use [file Supplementary_Table_1.pdf]

Supplementary Table 1: Between-case framework chart mapped across recovery stages, example quotes for early, mid and late stage recovery

**Superordinate theme**	**Categories**	**Theme**	**Early stage of recovery** **(0-6 months)**	**Mid stage** **(12 – 24 months)**	**Later stage** **(36 months+)**
Staying safe	gratitude	Gratitude	RON: glad to be here. I'm lucky to have a roof over my head. Thanks B (support group).	GARY: I love recovery and what it's given me.	LEN: Without recovery my life would be chaos and madness.
		Feeling lucky	LIZ: How lucky was I to be going on this (recovery training) course for free.	GARY: I'm very privileged (to get friendships back)	RON: so lucky, I've had a 2nd chance.
	Needing	Validation	LIZ: All of these people being clean and sober and part of a community: I love being with people.	TERRY: I want to be part of it (being with others in recovery)	TERRY: my daughter says (to me) 'don't drink cider' – you have to hang on to that.
		Belonging	GARY: it was nice because we were all there together.	LIZ: it's not easy being an ex-addict (corrects herself) – an addict.	LIZ: They are not like us (non-recovery people)
		Feeling scared	LIZ: I feel I was quite fragile (in the early stage)	BARBARA: for them (work assessors) to tell me if I'm well enough to work (made me) really anxious (doesn't feel up to working yet but will lose benefits).	RON: I'm not going to risk a 3rd chance. I'm staying where I am.

Exploring	Taking risks	Feeling exposed	LIZ: I felt a little anxious, if I'd understand it (the recovery training course)		LIZ: at times I have felt vulnerable – when you see yourself back, all the chinks in the armour... (implies surprise at surviving the recovery journey)
		Learning about self	BARBARA: the smile is real, loving it. (I'm) re-discovering life, finding myself.	GARY: I was basically scared... getting to know who I was again.	
		Helping others	LEN: I also give something back through sponsorship. I do a lot of voluntary work.	LEN: I now deliver a self-help group. That's a big step for me.	TERRY: I like helping people, I can point people in the right direction
	Seeking opportun- ities	frustration	GARY: I was getting told what to do (in the early stages)	TERRY: we're not all useless...	BARBARA: (feeling ill) I'm frustrated because I'm stuck in this flat. I hate it but I've got to take time out. I'm bored, bored, bored.
		Connecting With people	BARBARA: I come to groups to volunteer. It's so liberating.	RON: I've met a nice girl.. good luck to everyone.	TERRY: test the waters, in other places, not being scared.
	Integrity and purpose	Looking after self	GARY: (I learned) I was getting stronger in myself. Taking care of myself again. I learned there was nothing to be scared of.	RON: I hope everything pans out for everyone but I can only look out for myself and (my family).	BARBARA: asked myself a question: what does looking after (me) look like? So I said to the lady, 'I'm not coming in today,

Self-determining					I'm going to my friend's, do a bit of gardening which I enjoy'.
		Making own decisions		GARY: I made a brave decision to become what I wanted.	TERRY: recovery is when you are on your own making your own decisions. I'm getting the balls to say 'no'.
	Self worth and belief	Confidence	GARY: I used to do judo [ ] It felt good to be back (training). I felt a little something come back.	TERRY: bit by bit you get stronger. You get stronger as a person.	GARY: I spent 2 years of my life being unhappy and I wasn't prepared to sacrifice my happiness any more.
		Pride and respect		GARY: I've done it. Able to keep a job, be reliable, freedom from pain.	TERRY: Getting myself right. I'm not getting any help ... doing it myself.
	'The real you'	Acceptance		RON: I think I have become comfortably uncomfortable.	GARY: for me, all the pain, all those mistakes were worth it.
		authentic		RON people talk to me at bus stops. That's a nice thing to have!	TERRY: 'recovery'. They should call it something else. It's not recovery, its living again.
