# Supplementary material for: ‘I’m getting the balls to say no’: Trajectories in long-term recovery from problem substance use
Source: J Health Psychol. 2020 Jul 21;27(1):69–80. doi: 10.1177/1359105320941248 (PMC8739601; doi:10.1177/1359105320941248)
Supplement: Supplementary_Table_2 – Supplemental material for ‘I’m getting the balls to say no’: Trajectories in long-term recovery from problem substance use [file Supplementary_Table_2.pdf]

Supplementary Table 2: Within case framework chart– example from TERRY

	Staying safe		exploring		Self-determination		
**Stage of recovery**	**Gratitude**	**Needing**	**Taking risks**	**Seeking/needing opportunities**	**Self worth and belief**	**Integrity and purpose**	**The real you**
**Early**	(treatment centre) they were fantastic	People care about you, because nobody cared about me. I've come to meet people.<br>I need a bit of direction (about work)		I was an ordinary working person, you get stuck in a ball, you do certain things		(I had) no purpose in life (before recovery).	
**Mid**	(before recovery) nobody there to listen to us	We need people to help us along the trip – we will fall. Not be pushed, be there for us.	I want to be part of it (helping others)	We're not all useless.			
**Later**		Daughter says (to me), 'don't drink cider' you have to hang on to that.		I like helping people – I can point people in the right direction	Getting myself right (for health). Not getting any help, I do it myself.	Recovery is when you are on your own making your own decisions, I'm getting the balls to say 'no'	Getting myself back.<br>'Recovery', they should call it something else – it's not recovery, it living again
